# Supplementary material for: Molecular Biomarkers in Glioblastoma: A Systematic Review and Meta-Analysis
Source: Int J Mol Sci. 2022 Aug 9;23(16):8835. doi: 10.3390/ijms23168835 (PMC9408540; doi:10.3390/ijms23168835)
Supplement: Supplementary file 1 [file ijms-23-08835-s001.zip › ijms-1833492-supplementary.pdf]

Supplementary Table S1: Overall Survival sensitivity analysis of MGMT methylation

**Alkylating agents**	**HR (95% CI)**	**Test for overall effect: Z (p-value)**
Han 2014	1.67 [1.21,2.30]	3.12 (0.002)
Jan 2018 reference group	1.72[1.40,2.13]	5.06 (<0.00001)
Roodakker 2016 < 60years	1.59 [1.10,2.28]	2.48 (0.01)
Roodakker 2016 > 60years	1.68 [1.16,2.43]	2.75 (0.006)
Tini 2015	1.52 [1.10,2.11]	2.52 (0.01)
Weller 2015	1.52 [1.07,2.16]	2.36 (0.02)
**All included**	**1.66 [1.23,2.18]**	**3.40 (0.0007)**
**Tyrosine kinase inhibitors**		
Butowski 2011	1.63 [1.20,2.21]	3.12 (0.002)
Cloughesy 2017	1.69 [1.15,2.47]	2.68 (0.007)
Erdem Eraslan 2016	1.90 [1.16,3.13]	2.55 (0.01)
Omuro 2014	1.97 [1.40,2.77]	3.90 (<0.0001)
Reardon 2018	1.80 [1.20,2.71]	2.84 (0.005)
Reardon 2020 Bevacizumab arm	1.85 [1.14,2.98]	2.59 (0.01)
Wirsching 2018	1.98 [1.29,3.04]	3.12 (0.002)
**All included**	**1.82 [1.25,2.64]**	**3.16 (0.002)**

HR and z-values represent the values obtained after removing the indicated study from the overall MGMT methylation subgroup analysis. Values in bold represents the HR and z-value including all the studies in the subgroup analysis.

Supplementary Table S2: Overall Survival sensitivity analysis of IDH1 mutation

**Study ID**	**HR (95% CI)**	**Test for overall effect: Z (p-value)**
Collins 2014	2.63 [1.29,5.34]	2.67 (0.008)
Erdem Eraslan 2016	2.46 [1.58,3.84]	3.97 (<0.0001)
Jan 2018 DCTA group	2.40 [1.83,3.15]	6.29 (<0.00001)
Jan 2018 Reference group	2.35[1.78,3.09]	6.10 (<0.00001)
Latsch 2013	2.28 [1.72,3.02]	5.77 (<0.00001)
Reardon 2018	2.45 [1.71,3.53]	4.83 (<0.00001)
**All included**	**2.37 [1.81,3.12]**	**6.21 (<0.00001)**

HR and z-values represent the values obtained after removing the indicated study from the overall MGMT methylation subgroup analysis. Values in bold represents the HR and z-value including all the studies in the subgroup analysis.

Supplementary Table S3: Overall Survival sensitivity analysis of EGFR amplification or overexpression

Study ID	HR (95% CI)	Test for overall effect: Z (p-value)
Batchelor 2013	1.26 [0.92,1.74]	1.42 (0.15)
Abdullah 2015	1.22 [0.090,1.66]	1.29 (0.20)
Michaelson 2013	1.45 [0.94,2.24]	1.66 (0.10)
Srividya 2010	1.48 [1.08,2.02]	2.42 (0.02)
Tini 2015	1.17 [0.89,1.55]	1.11 (0.27)
**All included**	**1.31 [0.97,1.78]**	**1.74 (0.08)**

HR and z-values represent the values obtained after removing the indicated study from the overall MGMT methylation subgroup analysis. Values in bold represents the HR and z-value including all the studies in the subgroup analysis.
